# Influence of Arabic Gum/Gelatin/Ascorbyl Palmitate Coating on Quality Parameters of Hazelnut Kernels Stored in Plastic Boxes

**DOI:** 10.3390/molecules30204126

**Published:** 2025-10-19

**Authors:** Dariusz Kowalczyk, Katarzyna Niedźwiadek, Tomasz Skrzypek, Emil Zięba, Jaromir Jarecki

**Affiliations:** 1 Department of Biochemistry and Food Chemistry, Faculty of Food Sciences and Biotechnology, University of Life Sciences in Lublin, Skromna 8, 20-704 Lublin, Poland; katarzyna.lupina@gmail.com; 2 Department of Biomedicine and Environmental Research, Institute of Biological Sciences, Faculty of Medicine, The John Paul II Catholic University of Lublin, Konstantynów 1 J, 20-708 Lublin, Poland; tomasz.skrzypek@kul.pl (T.S.); emil.zieba@kul.pl (E.Z.); 3 Department of Traumatology, Orthopedics and Rehabilitation, Medical University of Lublin, 20-954 Lublin, Poland; jaromir.jarecki@umlub.pl

**Keywords:** hazelnuts, edible coating, ascorbyl palmitate, storage, antioxidant activity, rancidity

## Abstract

Edible coatings enriched with antioxidants offer a promising approach to prolong the shelf life of oxidation-sensitive foods such as nuts. Nonetheless, not all formulations provide the expected protection, and understanding why is equally important. The aim of this study was to assess the effect of an Arabic gum/gelatin/ascorbyl palmitate (GAR/GEL/AP) coating on the quality of hazelnut kernels during storage at 23 °C and ~40% relative humidity. The coating was applied by dipping hazelnuts in a 20% ethanolic solution containing GAR/GEL 75/25 blend (10% *w*/*w*), glycerol (1% *w*/*w*), Tween 80 (0.25% *w*/*w*), and AP (2% *w*/*w*), followed by drying. Control (uncoated) and coated hazelnuts were stored in plastic containers and evaluated at 1, 2, 4, 8, and 16 weeks for weight loss, moisture content, hardness, color, 2,2-diphenyl-1-picrylhydrazyl radical (DPPH*) scavenging activity, acid and peroxide values, and thiobarbituric acid reactive substances (TBARS) level. Coated hazelnuts showed higher initial moisture content (8.17%), stabilizing at 4.80% after one week, compared to 3.35% in uncoated samples. This increased moisture led to greater storage-related weight loss. The coating darkened the nuts and reduced their yellow hue. It had no significant effect on hardness, peroxide value, or TBARS index, but notably enhanced the antiradical potential. After 16 weeks, coated nuts had an acid value ~10 mg KOH/g lower than the control. In conclusion, the coating improved antioxidant capacity and reduced hydrolytic, but not oxidative, rancidity in hazelnuts. Therefore, further optimization of the coating formulation or application method is necessary to more effectively improve the shelf life of hazelnuts.

## 1. Introduction

Reducing food waste is a critical priority within the European Union, supported by regulatory frameworks aimed at enhancing sustainability and efficiency across the food system [1]. These regulations promote innovation in food preservation methods to extend shelf life, improve product quality, and reduce losses along the supply chain, particularly at the household level, where a substantial share of food waste occurs [2]. One of the main factors contributing to food quality deterioration is oxidation, which can reduce nutritional value, alter sensory attributes, and lead to the formation of harmful compounds. To counteract these effects and prolong shelf life, a variety of protective strategies have been developed. Among them, biopolymer-based edible coatings have gained increasing attention. These coatings can perform functions similar to conventional packaging by acting as semi-permeable barriers to oxygen and moisture, thereby slowing lipid oxidation and limiting moisture absorption or desorption. Such properties make the coatings especially useful for oxidation-sensitive products like nuts.

Incorporating antioxidants into coating formulations can further enhance their protective efficacy [3]. The effectiveness of these active coatings depends on the type and concentration of antioxidants, as well as on interactions between coating components, antioxidants, and the food matrix, which influence the release and migration of active agents to target sites. Careful selection of antioxidants must therefore take into account their polarity, mechanism of action, and compatibility with the biopolymer matrix. According to the ‘polar paradox’ theory, hydrophilic antioxidants are more effective in non-polar environments (e.g., pure oils), whereas lipophilic antioxidants perform better in polar systems such as emulsions, liposomes, biological membranes, or tissues [4]. This suggests that the polarity of antioxidants should be matched with the food matrix to achieve optimal activity. Since nuts have low moisture (typically below 5%) and high fat content (around ≥60%) [5,6], vitamin C (a polar antioxidant) would be expected to act effectively as an antioxidant in coatings. However, a previous study [7] showed that the incorporation of L-ascorbic acid (AA) into the coating formulation did not enhance the antiperoxidant properties when applied to walnuts.

This discrepancy indicates that the paradigm of the polar antioxidant paradox oversimplifies the mechanisms of antioxidant activity [4]. The framework primarily classifies antioxidants as either polar or nonpolar and does not explicitly account for amphiphilic molecules. Ascorbyl palmitate (AP) is an amphiphilic derivative of vitamin C, combining a hydrophilic ascorbate moiety with a lipophilic palmitoyl chain. Its amphiphilic character allows AP to interact with both aqueous and lipid phases, potentially facilitating distribution at oil–water interfaces. The antioxidant efficiency of AP depends on its release from the biopolymer matrix [8] and its availability to lipid-rich food components; however, its extremely low water solubility (0.00003 g/L at 25 °C) and unknown solubility in oils may limit its distribution within coatings and, consequently, its effectiveness in protecting the nut lipids. Compared to AA, AP is more stable. When incorporated into the film-forming formulation, the hydrophobic palmitoyl chain improves the water vapor barrier properties of biopolymer-based materials [8,9], effectively minimizing moisture loss or uptake during storage [10], which makes AP a promising antioxidant for active edible coatings. So far, AP has not been widely used for this purpose; it has only been applied in combination with α-tocopherol in whey protein-based edible coatings, but such coatings, with or without antioxidants, showed similar effects in slowing lipid oxidation in roasted peanuts [11].

Due to its partial hydrophobicity and high melting point (107–117 °C), AP is difficult to emulsify in aqueous coating systems, necessitating the use of co-solvents and emulsifiers [12]. Therefore, incorporating amphiphilic biopolymers into coating formulations is advantageous, as they can serve simultaneously as carriers, emulsifiers, and stabilizers. Blending different biopolymers and/or surfactants/emulsifiers can enhance their emulsifying performance through synergistic interactions between components. Gum Arabic (GAR) is a highly branched heteropolysaccharide with hydrophilic sugar groups and some amphiphilic protein moieties, enabling it to bridge oil and water by forming a protective film around oil droplets and preventing coalescence. Gelatin (GEL), a protein containing both hydrophilic and hydrophobic amino acids, provides additional surface-active properties that help stabilize emulsions. Although GEL’s emulsifying ability is generally weaker than that of GAR, its gelling and thickening properties help maintain the stability of oil–water emulsions during film formation [13,14].

Building on these considerations, this study selected a GAR/GEL formulation as a carrier for AP based on prior research [8] showing that a 75/25 GAR/GEL blend provides the slowest, most controlled AP release and prolonged antioxidant activity. The branched structure of gum Arabic likely helps trap AP, limiting its migration and sustaining its antioxidant action on product surface, where oxidative processes are most intense.

The current work applies the GAR/GEL/AP system to hazelnuts (high-fat, low-moisture food) to evaluate its effectiveness in extending shelf life and protecting product quality. The objective of this study was to assess how a GAR/GEL/AP edible coating influences the key quality parameters (weight loss, moisture content, texture, color, antioxidant activity, and markers of lipid degradation) of hazelnut kernels during 16 weeks of storage in at 23 °C and 40 ± 5% relative humidity in closed polypropylene boxes, in the absence of light.

## 2. Results

### 2.1. Characterization of Coating-Forming Emulsion (CFE)

**Figure 1:**
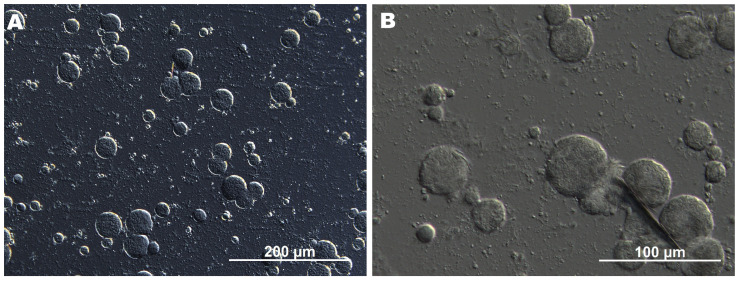
Arabic gum/gelatin/ascorbyl palmitate coating-forming emulsion under differential interference contrastmicroscopy at 200× (**A**) and 600× (**B**) magnification.

DIC microscopy revealed that the CFE consisted of spherical droplets with well-defined interfaces and a relatively uniform circular morphology (Figure 1). The droplet size ranged approximately from 6 to 40 μm, indicating effective emulsification and moderate size homogeneity. This relatively narrow size distribution and the lack of irregular structures suggest that the emulsion was stable, with only limited signs of coalescence or agglomeration. In contrast, such an effect was not observed in a previous study, where AP in the GAR-based emulsion predominantly appeared as large acicular crystals [8]. This highlights the improved dispersion and stabilization achieved in the current formulation through ultrasonic processing. At higher magnification, needle-like AP crystals, with an average length of 4–11 μm, were observed inside the emulsion droplets, while the continuous phase contained only dispersed microparticles (Figure 1B). This localization may be explained by hydrophobic interactions between AP, likely enhanced by the presence of Tween 80 and ethanol, which reduced interfacial tension and facilitated the incorporation of water-insoluble AP. Also, the emulsifying properties of biopolymers (GAR and GEL) may have further promoted internal organization and retention of AP within the droplets. This mechanism is consistent with previous findings in polyethylene glycol–water systems, where AP was shown to form crystalline or liquid crystalline mesophases, suggesting a tendency toward self-assembly under certain conditions [15].

The pH of the CFE was 5.10 (Table 1), slightly higher than reported in a previous study [8], possibly due to the previously mentioned variations in the emulsion microstructure. Measuring the pH of emulsions is inherently challenging because of the coexistence of oil and water phases and the likelihood of heterogeneous pH distribution within the system. It is worth mentioning that the pH value of the CFE was close to that of hazelnuts surface (Table 2), which may be beneficial for preserving the sensory and chemical stability of the product while avoiding the excessive acidity typically introduced by formulations containing AA [16].

The η of the CFE was 48.50 ± 5.87 mPa·s (Table 1), indicating a fluid with moderate viscosity. According to visual observations, this level of viscosity provides sufficient thickness to support uniform coating formation without runoff, while maintaining adequate fluidity for practical application. Moreover, it may contribute to emulsion stability by minimizing phase separation during processing. Although the comparison should be interpreted with caution due to differences in formulation, under comparable η measurement conditions (25 °C, shear rate of 50 s^−1^), the viscosity of the current emulsion is higher than that reported for pea protein-based emulsions containing 2% candelilla wax or oleic acid (≈10 mPa·s) [17]. This may be attributed to the presence of GEL, which enhances the structuring of the continuous phase and promotes network formation.

### 2.2. Microstructure of Hazelnuts

**Figure 2:**
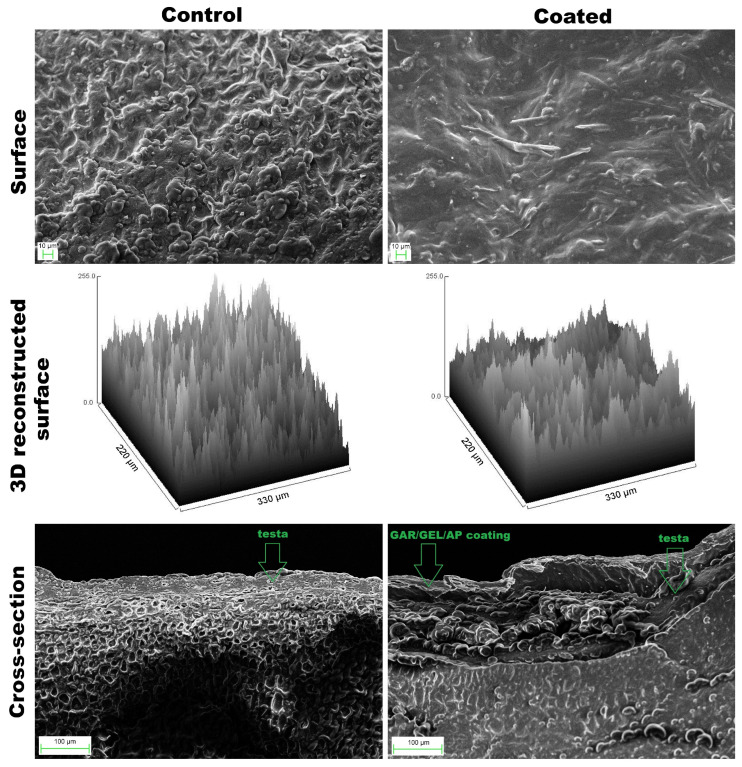
Scanning electron microscopy images of the surface (1000×), 3D reconstructed surface, and cross-section (500×) of control (uncoated) and Arabic gum/gelatin/ascorbyl palmitate emulsion-coated hazelnut kernels.

The surface of uncoated hazelnuts was characterized by a rough and irregular topography (Rq = 57.57), whereas the coated hazelnuts exhibited a smoother and more homogeneous surface morphology (Rq = 40.22, Figure 2, Table 2). Needle-like AP crystals, accompanied by much smaller globules, were abundantly distributed across the surface. It can be observed that they were well-developed and noticeably larger than those in CFE (Figure 1), likely as a result of co-solvent (ethanol) evaporation. 

**Figure 3:**
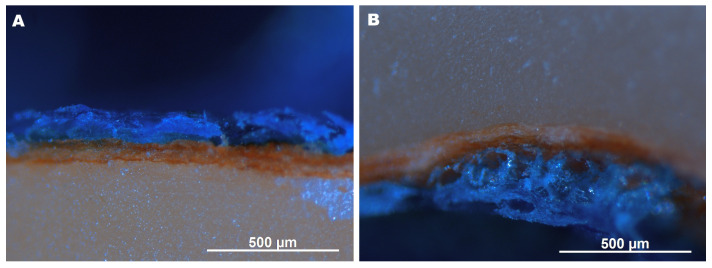
Cross-sectional images (100×) of Arabic gum/gelatin/ascorbyl palmitate emulsion-coated hazelnut kernels, stained with methylene blue, showing the upper (**A**) and the lower (**B**) part of the nut.

In the cross-sectional view, the testa (skin) appeared as a thin outer layer with non-uniform thickness (Figure 2 and Figure 3). Dipping in the emulsion formed a continuous, loose coating on the testa. Partial detachment in some areas, likely due to mechanical sectioning, suggests weak adhesion of the coating. As confirmed by light microscopy observations (Figure 3), no impregnation of the testa and nut interior with the emulsion occurred, as evidenced by the absence of blue coloration within the cross-section. The coating was uneven, tending to be visibly thicker at the bottom compared to the top (Figure 3, Table 1). The coating thickness was similar to or exceeded that of the testa (Figure 2 and Figure 3).

### 2.3. pH, Weight Loss (WL), Moisture Content (MC), Hardness, and Color Parameters

**Figure 4:**
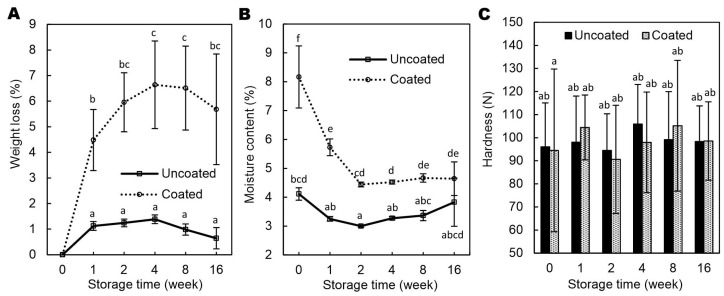
The effect of Arabic gum/gelatin/ascorbyl palmitate coating on weight loss (**A**), moisture content (**B**), and hardness (**C**) of hazelnut kernels stored in polypropylene boxes at 23 °C and ~40% relative humidity. Values with different superscript letters (a–f) are significantly different (*p* < 0.05).

Since the CFE had a pH of 5.10 (Table 1), the surface pH of the coated hazelnuts was lower than that of the control (5.14 vs. 5.60; Table 2), while the pH of the interior surface remained unaffected by the coating. This proves that CFE did not penetrated through hazelnut skin (Figure 3). Nevertheless, hazelnuts absorbed moisture during coating, as indicated by the data in Figure 4.

As shown in Figure 4A, coated nuts exhibited greater WL in comparison to the uncoated samples (4.48–6.64 vs. 0.64–1.38%), which was attributed to their higher initial MC (8.17% vs. 3.35%; Figure 4B) resulting from moisture uptake. The elevated MC enhanced the moisture gradient, thereby accelerating water migration over time. Similar results were previously observed for walnuts coated with emulsion-based coatings [7]. Throughout storage, the coated hazelnuts retained significantly more moisture than the noncoated ones, suggesting that the GAR/GEL/AP coating acted as a barrier to water loss and effectively mitigated dehydration; however, by the end of the 16-week period, the differences in MC were no longer statistically significant. As commonly known, low MC in nuts is crucial primarily to inhibit microbial growth and reduce the risk of aflatoxin contamination [18,19]. It should be noted that, after one week of storage, the MC in the coated hazelnuts was below 6% (Figure 4B), which complies with the European Union limit of 7% for hazelnut kernels [20] and also aligns with the commonly recommended maximum of 6% for optimal storage stability [21,22].

As shown in Figure 4C, no significant differences (*p* < 0.05) in hardness were observed between coated and uncoated hazelnuts during storage. This suggests that moisture uptake (Figure 4B) did not negatively affect kernel texture, which is beneficial from a sensory perspective. The hardness of hazelnuts varied between approximately 90 and 106 N, with standard deviations of 14–35 N, consistent with variability reported in previous studies [23,24]. The 16-week storage had no significant impact on hardness, contrary to Razavi et al. [25], who observed increasing rupture force over time. In contrast, Correia et al. [26], found that high humidity reduced hardness and friability, while Guinée et al. [24] noted increased friability of hazelnut kernels only at 90% relative humidity without packaging. These findings indicate that storage effects on hazelnut texture depend strongly on environmental factors such as humidity and packaging.

**Figure 5:**
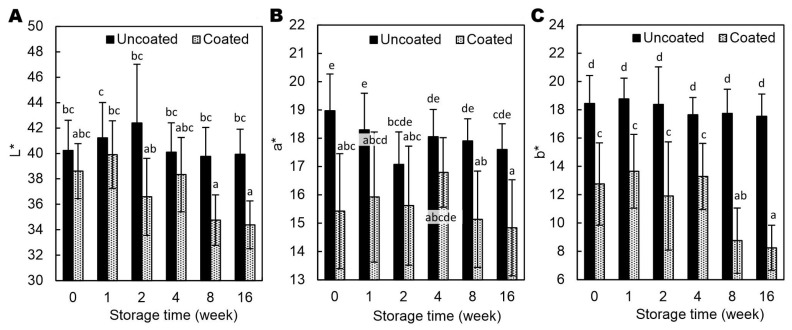
The effect of Arabic gum/gelatin/ascorbyl palmitate coating on lightness (L*) (**A**), redness (a*) (**B**), and yellowness (b*) (**C**) of hazelnut kernels stored in polypropylene boxes at 23 °C and ~40% relative humidity. Values with different superscript letters (a–e) are significantly different (*p* < 0.05).

From week 8, the coated hazelnuts showed noticeable darkening compared to initial values and to uncoated samples (Figure 5A). Unlike walnuts coated with AA-added emulsion, which lighten due to strongly lowered pH (4.37) [7,16], the AP used in this study only slightly reduced the surface pH (Table 2) and thus did not bleach the polyphenolic pigments. The coated hazelnuts showed lower b* values, indicating reduced yellowness, with a tendency toward lower a* values as well, suggesting less redness (Figure 5B,C). It is possible that the coating partially masks the natural color of the hazelnut skin by reducing light reflection and increasing diffuse scattering by AP crystals (Figure 2), leading to a duller, less saturated appearance.

A two-way ANOVA, based on the F-test, revealed significant interaction effects between treatment (coated vs. uncoated kernels) and storage time for several physicochemical parameters of hazelnuts. In particular, WL (*p* < 0.05), MC (*p* < 0.001), lightness (*p* < 0.05), and yellowness (*p* < 0.005) (Table S1) exhibited treatment-dependent changes throughout the storage period, indicating that the applied coating influenced the rate and extent of moisture loss and color development. In contrast, hardness and redness a* (*p* > 0.05) showed no significant interaction, suggesting that these properties followed a comparable trend over time irrespective of treatment.

### 2.4. Antiradical Activity, Acid Value (AV), Peroxide Value (PV), and Thiobarbituric Acid Reactive Substances (TBARS) Concentration

**Figure 6:**
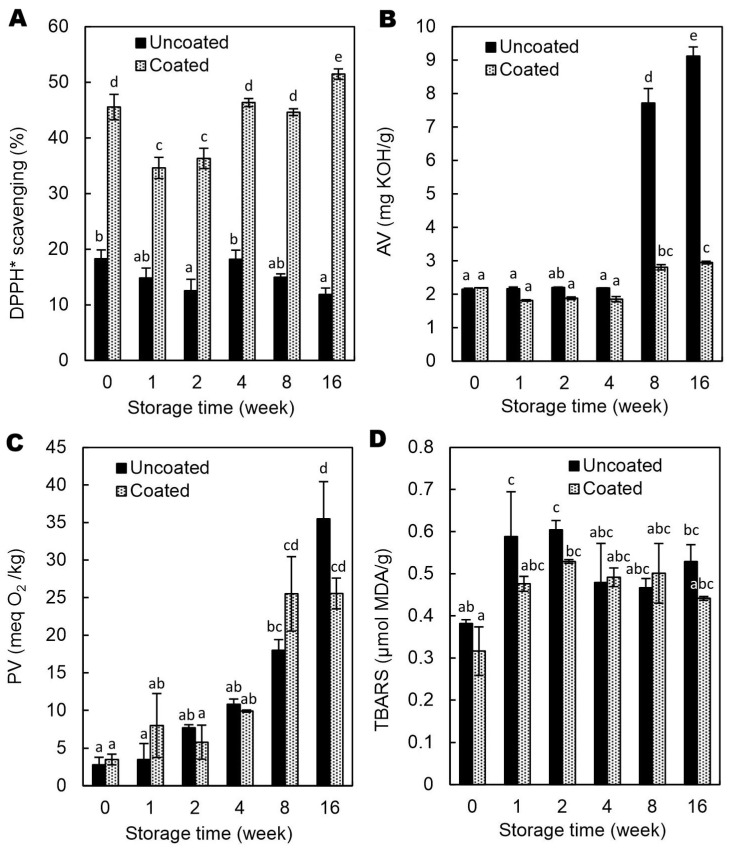
Effect of Arabic gum/gelatin/ascorbyl palmitate coating on the DPPH* radical scavenging activity of hazelnut kernels (**A**) stored in polypropylene containers at 23 °C and ~40% relative humidity, as well as on acid value (AV) (**B**), peroxide value (PV) (**C**), and thiobarbituric acid reactive substances (TBARS) concentration (**D**) in oil from these hazelnuts. Values with different superscript letters (a–e) are significantly different (*p* < 0.05). meq—milliequivalent, MDA—malondialdehyde.

Hazelnuts are a rich source of bioactive compounds such as phenolic acids, proanthocyanidins, and tocopherols, which contribute to their significant antioxidant activity and associated health benefits [27,28]. Accordingly, the 2,2-diphenyl-1-picrylhydrazyl radical (DPPH*) scavenging activity of uncoated hazelnuts (Figure 6A) was likely attributed to the synergistic action of alcohol-extractable polyphenols, including esterified gallic acid and condensed tannins concentrated in the skin [29] and α-tocopherol [30]. The GAR/GEL/AP-coated hazelnuts exhibited 2.5 to 4.5-fold increase in antiradical activity compared to the control (Figure 6A). Considering the 2% AP concentration in CFE, the observed increase is relatively small and likely limited by the coating thickness (~100 μm, Table 1), small surface area, and one-sided contact (film studies typically allow contact with the free radical on both sides). Moreover, previous comparative studies [8] have shown that this type of material exhibits the slowest release rate of AP, suggesting limited migration of the antioxidant from the coating to the acceptor medium with the free radicals. The DPPH* scavenging activity, assessed over time using an immersion method with whole kernels, showed some variability (Figure 6A), likely reflecting differences in surface contact, and thus in the rate of AP release. While this method provides a reliable measure of surface antioxidant activity, it primarily captures the coating’s immediate effects and may not fully reflect potential synergistic interactions between the coating components and the hazelnut during consumption.

The AV, indicating lipase-induced hydrolytic degradation, was similar in oils from uncoated and coated hazelnuts during the first 4 weeks (Figure 6B). After 8 weeks, AV increased sharply, yet remained significantly lower in the coated samples (*p* < 0.05). One possible explanation is that the CFE, which contained 20% ethanol, reduced the activity of lipases on the kernel surface. Furthermore, ethanol likely inactivated most molds [31] and, consequently, their lipases, which are known to contribute synergistically to the hydrolytic rancidity of nuts [32]. While further studies are needed to confirm this mechanism, existing literature provides supporting evidence that is consistent with this hypothesis. Gull et al. [33] reported that sodium alginate coatings enriched with β-tocopherol significantly reduced free fatty acid content (0.9% vs. 1.4% oleic acid) and microbial counts (1.9 vs. 3.2 log CFU/g) in fresh walnuts compared to uncoated samples [33]. According to the authors, the reduction in microbial growth could be due to limited oxygen supply by creating a barrier. Similarly, other studies have reported that coatings containing different bioactive compounds effectively reduced microbial, including fungal, growth on nut surfaces by providing antimicrobial protection [34,35,36].

Given that the endogenous lipases of intact nuts are generally inactive on the surface [37], the coating-induced decrease in surface pH (Table 2) was unlikely to have contributed significantly to the observed reduction in hydrolytic activity. Notably, in a previous study [7], coating containing AA applied to walnuts resulted in lower AV compared to the control and AA-free coated samples, likely due to the low pH of the formulation, which could have influenced lipase activity.

The coating did not prevent oxidative deterioration of oil in hazelnuts, as indicated by peroxide and TBARS values (Figure 6C,D). This limited effectiveness is likely related, at least in part, to the coating’s microstructure: surface irregularities, micropores, and weak adhesion to the kernel (Figure 2 and Figure 3), that reduced its barrier function against oxygen. Additionally, AP represents a specific case among antioxidants; despite being amphiphilic, it shows almost no solubility in water or oil at room temperature. This dual character may hinder its localization at lipid oxidation sites, explaining its negligible effect on the PV and TBARS. These findings challenge the assumption that edible coatings consistently provide antioxidative protection, highlighting that their efficacy strongly depends on composition, structure, and substrate interactions.

The F-test showed that all rancidity-related parameters were significantly affected by the interaction between treatment and storage time. Specifically, DPPH* scavenging activity (*p* < 0.005), AV (*p* < 0.001), PV (*p* < 0.05), and TBARS (*p* < 0.05) (Table S1) demonstrated distinct patterns, indicating that antioxidant capacity and rancidity processes evolved differently depending on whether kernels were coated or uncoated.

## 3. Materials and Methods

### 3.1. Materials

Shelled hazelnut kernels (*Corylus avellana* L.) were purchased from a commercial supplier in Lublin, Poland. For the study, only kernels weighing approximately 1.5 g each and free from visible defects, discoloration, or signs of spoilage were selected. GAR Agri-Spray Acacia R (Agrigum International, High Wycombe, UK) and pork GEL (bloom strength 240; McCormick-Kamis, Wólka Kosowska, Poland) were used as coating-forming biopolymers. Glycerol, AP, Tween 80, and 2,2-diphenyl-1-picrylhydrazyl (DPPH) were obtained from Sigma Chemical Co. (St. Louis, MO, USA). Ethanol (99.8%) was purchased from Avantor Performance Materials Poland S.A. (Gliwice, Poland).

### 3.2. Coating Formulation and Procedure

The coating-forming emulsion (CFE) was prepared following the procedure described by Łupina et al. [8], with the modification of using ultrasound emulsification as described previously [38]. The CFE consisted of a 20% aqueous-ethanolic solution containing GAR/GEL 75:25 blend (10% *w*/*w*), glycerol (1% *w*/*w*), Tween 80 (0.25% *w*/*w*), and AP (2% *w*/*w*). Sonication was performed using a Tefic 1800 W probe sonicator with a 20 mm diameter probe (TEFIC BIOTECH CO., Ltd., Xi’an, China) at 80% amplitude. The process involved two 1 min intervals in a pulsed mode (2 s on, 2 s off) with a 30 s pause between them, resulting in a maximum temperature of 70 °C. The CFE was subsequently degassed using a stainless steel mesh sieve. The kernels skewered on sword toothpicks were immersed in the CFE for 30 s and then air-dried for 1 h. Uncoated hazelnut kernels were used as the control. The uncoated and coated hazelnuts were packed in portions of 10 pieces per container into 200 mL polypropylene PRATIPACK boxes (107 mm in width, 80 mm in length, and 40 mm in height; Guillin Polska, Oleśnica, Poland), sealed with lids, and stored under controlled conditions (23 ± 1 °C, 40 ± 5% relative humidity) in a closed cabinet, completely protected from light. The samples for analysis were collected at week 0 (baseline), and after 1, 2, 4, 8, and 16 weeks of storage.

### 3.3. Analysis of CFE

To examine the morphology of CFE, a Leica 5500B microscope (Leica Microsystems GmbH, Wetzlar, Germany) with differential interference contrast optics was used. The pH of the CFE was measured at 30 ± 1 °C using a flat surface electrode (Elmetron EPX-3, Zabrze, Poland) connected to a pH meter (Elmetron CPC 401, Zabrze, Poland). The dynamic viscosity (η, mPa·s) of CFE at 30 ± 1 °C was assessed using a ROTAVISC lo-vi rotational viscometer (IKA, Staufen, Germany) equipped with a VOLS-1 adapter and a VOL-SP-6.7 spindle, operating at 50 rpm for 2 min, with a sample volume of 6.7 mL. All measurements were conducted in triplicate.

### 3.4. Microstructure

The microstructure of the surface and cross-sections of the hazelnuts was analyzed using a scanning electron microscope (1430VP, LEO Electron Microscopy Ltd., Cambridge, UK). The film samples were re-dried under vacuum and coated with gold before observation. ImageJ version 1.54j open-source software was used to reconstruct 3D surface profiles from 2D topographical images and to determine surface roughness (Rq) [39]. Additionally, the thickness of the coating stained with methylene blue was measured from microscopic images captured using a Leica 205C microscope equipped with a Leica DFC500 camera (Leica Microsystems, Wetzlar, Germany) and analyzed with AxioVision 4.8 software (Carl Zeiss, Jena, Germany). Longitudinal cross-sections of the coated hazelnuts were prepared using a scalpel, and the coating thickness was measured at the top, lateral, and bottom regions through microscopic analysis.

### 3.5. pH

The pH of the surface and cross-sections of the hazelnuts was measured as described in Section 3.3, after slight surface hydration with 20.0 μL of deionized water.

### 3.6. Determination of Weight Loss (WL), Color Parameters, Hardness, and Moisture Content (MC)

WL was measured on four replicate boxes per treatment by repeatedly weighing the same entire batch of hazelnut kernels in each box and calculating the percentage decrease compared to the initial weight (~15 g).

Color values (L*, a*, b*) were measured with a colorimeter (NH310, 3nh, Guangzhou, China) on 10 kernels per treatment, each randomly taken from different boxes. The same kernels were then used for hardness testing, performed with a TA-XT2i texture analyzer equipped with a 50 kg load cell (Stable Micro Systems, Godalming, UK). Kernels were compressed using a 100 mm diameter platen at a constant speed of 5 mm/s until a penetration depth of 2 mm was reached. After hardness analysis, the remaining was pooled, ground, and approximately 2 g of the sample was dried at 105 °C for 24 h, in four replicates. MC was calculated as the percentage of weight loss after drying.

### 3.7. Determination of Antiradical Properties

The antiradical activity of hazelnut kernels was assessed using the DPPH* scavenging assay. A single kernel (~1.5 g) was placed in contact with 20 mL of a methanolic DPPH* solution (absorbance of 2.5 ± 0.05 at 517 nm, 23 °C). The solution was mixed using a magnetic stirrer (400 rpm) and circulated through a flow-through cuvette (Lambda 40 spectrophotometer, Perkin–Elmer, Norwalk, CT, USA) via a peristaltic sipper pump (“PESI” B2190036, Perkin–Elmer, Norwalk, CT, USA). A perforated barrier was placed between the kernel and the stir bar. Absorbance at 517 nm was continuously monitored for 1 h. The results were expressed as the percentage of DPPH* neutralized, calculated using the Formula (1):

DPPH* scavenging (%) = [(A_0_ − A_s_)/A_0_] × 100 (1)

where A_0_ is the initial absorbance of the DPPH* solution, and A_s_ is the absorbance of DPPH* solution after 1 h contact with the hazelnut. The measurements were performed in triplicate, using one kernel randomly taken from each of three independent boxes.

### 3.8. Determination of Acid Value (AV), Peroxide Value (PV), and Thiobarbituric Acid Reactive Substances (TBARS) Concentration in Hazelnut Oil

Lipid extraction was carried out according to the previously described method [7]. The AV and PV for the extracted oil were determined according to [40] ISO 660:2020 and [41] ISO 3960:2017, respectively. TBARS values were measured following the procedure outlined by [42]. All measurements were performed in triplicate.

### 3.9. Statistical Analysis

All results were expressed as mean ± standard deviation (n ≥ 3). Statistical differences among mean values were evaluated using two-way analysis of variance (ANOVA), with treatment and storage time as fixed factors. The F-test was applied to evaluate the presence of interaction effects between the studied factors, after which Tukey HSD post hoc test was performed using Statistica software, version 13.3 (TIBCO Software Inc., Palo Alto, CA, USA). Differences were considered statistically significant at *p* < 0.05.

## 4. Conclusions

The GAR/GEL/AP coating doubled the moisture content of hazelnuts, which led to greater weight loss during storage, highlighting the need for more efficient post-coating drying. Although the coating enhanced antioxidant (health-promoting) properties, it did not provide full oxidative protection, likely due to insufficient barrier integrity and adhesion, possibly caused by emulsion destabilization during co-solvent (ethanol) evaporation and subsequent AP recrystallization. The observed reduction in hydrolytic rancidity may result from ethanol effects on endogenous and/or fungal lipases; however, lipase activity was not measured in this study, and further investigations are needed to confirm this mechanism.

Overall, the coating significantly influenced storage-related changes in hazelnut quality. While hardness and redness were unaffected, parameters such as WL, MC, lightness, yellowness, antiradical activity, AV, and oxidative deterioration were strongly affected by the interaction between coating type and storage duration. This emphasizes that coating efficacy depends on both composition and storage conditions, underlining the importance of selecting appropriate coatings and monitoring storage to maintain product quality.

Further optimization, such as improving emulsion homogeneity, adjusting ingredient ratios, or applying multilayer coatings, is needed to reduce moisture uptake and enhance oxygen barrier performance, ultimately achieving a more uniform, less porous, and better-adhering coating layer.

## Figures and Tables

**Table 1:** pH and dynamic viscosity (η) of Arabic gum/gelatin/ascorbyl palmitate coating-forming emulsion (CFE). The thickness of coating formed on hazelnut kernels.

Parameter	Value
pH of CFE	5.10 ± 0.03
η of CFE (mPa·s)	48.50 ± 5.87
Coating thickness at the top (μm)	93.11 ± 23.23
Coating thickness at the bottom (μm)	112.71 ± 76.27

**Table 2:** The surface root-mean-square roughness (Rq) and pH of the outer and inner surfaces of control (uncoated) and Arabic gum/gelatin/ascorbyl palmitate emulsion-coated hazelnut kernels.

Parameter	Control	Coated
Rq	57.57± 10.37 ^b^	40.22 ± 6.13 ^a^
pH of surface	5.60 ± 0.08 ^b^	5.14 ± 0.04 ^a^
pH of interior surface	6.09 ± 0.14 ^a^	6.10 ± 0.18 ^a^

Values with different superscript letters (a,b) in the row are significantly different (*p* < 0.05).

## Data Availability

The raw data supporting the conclusions of this article will be made available by the authors upon request.

## Supplementary Materials

The following supporting information can be downloaded at: https://www.mdpi.com/article/10.3390/molecules30204126/s1, Table S1: *p*-values from the F-test for the interaction effect between treatment (control and uncoated hazelnut kernels) and storage time for the analyzed dependent variables.

## Abbreviations

The following abbreviations are used in this manuscript:

a*	Red/Green coordinate
AA	Ascorbic Acid
AP	Ascorbyl Palmitate
AV	Acid Value
b*	Yellow/Blue coordinate
CFE	Coating-Forming Emulsion
DPPH*	2,2-Diphenyl-1-picrylhydrazyl radical
GAR	Gum Arabic
GEL	Gelatin
L*	Lightness
MC	Moisture Content
PV	Peroxide Value
Rq	Root-Mean-Square Roughness
TBARS	Thiobarbituric Acid Reactive Substances
WL	Weight Loss
η	Viscosity

## References

[1] European Commission EU Actions Against Food Waste. Food Safety—Food Waste. European Commission. https://food.ec.europa.eu/food-safety/food-waste/eu-actions-against-food-waste_en.

[2] European Commission Research and Innovation for Food Waste Prevention and Reduction at Household Level Through Measurement, Monitoring and New Technologies (HORIZON-CL6-2025-02-FARM2FORK-04-Two-Stage). CORDIS–EU Research Results. European Union. https://cordis.europa.eu/programme/id/HORIZON_HORIZON-CL6-2025-02-FARM2FORK-04-two-stage.

[3] Laguerre M., Bayrasy C., Panya A., Weiss J., McClements D.J., Lecomte J., Decker E.A., Villeneuve P. (2015). What Makes Good Antioxidants in Lipid-Based Systems? The Next Theories Beyond the Polar Paradox. Crit. Rev. Food Sci. Nutr..

[4] Mukwevho P.L., Kaseke T., Fawole O.A. (2025). Innovations in Biodegradable Packaging and Edible Coating of Shelled Temperate Nuts. Food Bioprocess. Technol..

[5] Gonçalves B., Pinto T., Aires A., Morais M.C., Bacelar E., Anjos R., Ferreira-Cardoso J., Oliveira I., Vilela A., Cosme F. (2023). Composition of nuts and their potential health benefits—An overview. Foods.

[6] U.S. Department of Agriculture, Agricultural Research Service (2019). FoodData Central. https://fdc.nal.usda.gov/.

[7] Kowalczyk D., Zięba E., Skrzypek T., Baraniak B. (2017). Effect of carboxymethyl cellulose/candelilla wax coating containing ascorbic acid on quality of walnut (*Juglans regia* L.) kernels. Int. J. Food Sci. Technol..

[8] Łupina K., Kowalczyk D., Drozłowska E. (2020). Polysaccharide/gelatin blend films as carriers of ascorbyl palmitate—A comparative study. Food Chem..

[9] Ortiz C.M., Salgado P.R., Dufresne A., Mauri A.N. (2018). Microfibrillated cellulose addition improved the physicochemical and bioactive properties of biodegradable films based on soy protein and clove essential oil. Food Hydrocoll..

[10] Galus S., Kadzińska J. (2015). Food applications of emulsion-based edible films and coatings. Trends Food Sci. Technol..

[11] Han J.H., Hwang H.M., Min S., Krochta J.M. (2008). Coating of peanuts with edible whey protein film containing α-tocopherol and ascorbyl palmitate. J. Food Sci..

[12] López-Martínez A., Rocha-Uribe A. (2017). Antioxidant hydrophobicity and emulsifier type influences the partitioning of antioxidants in the interface improving oxidative stability in O/W emulsions rich in n-3 fatty acids. Eur. J. Lipid Sci. Technol..

[13] Kowalczyk D., Baraniak B. (2014). Effect of candelilla wax on functional properties of biopolymer emulsion films—A comparative study. Food Hydrocoll..

[14] Kowalski Z., Banach M., Makara A. (2011). Otrzymywanie białka niskotemperaturowego mocno żelującego (żelatyny) metodami chemicznymi. Chemik.

[15] Benedini L., Messina P.V., Palma S.D., Allemandi D.A., Schulz P.C. (2012). The ascorbyl palmitate-polyethyleneglycol 400-water system phase behavior. Colloids Surf. B Biointerfaces.

[16] Kowalczyk D. (2016). Biopolymer/candelilla wax emulsion films as carriers of ascorbic acid—A comparative study. Food Hydrocoll..

[17] Kowalczyk D., Kazimierczak W., Zięba E., Lis M., Wawrzkiewicz M. (2024). Structural and physicochemical properties of glycerol-plasticized edible films made from pea protein-based emulsions containing increasing concentrations of candelilla wax or oleic acid. Molecules.

[18] Salvatore M.M., Andolfi A., Nicoletti R. (2023). Mycotoxin Contamination in Hazelnut: Current Status, Analytical Strategies, and Future Prospects. Toxins.

[19] Brar P.K., Danyluk M.D. (2018). Nuts and grains: Microbiology and preharvest contamination risks. Microbiol. Spectr..

[20] (2002). Commission Regulation (EC) No 1284/2002 of 15 July 2002 Laying Down the Marketing Standard for Hazelnuts in Shell. Official Journal of the European Communities L 182. https://eur-lex.europa.eu/legal-content/EN/TXT/?uri=CELEX:32002R1284.

[21] Turan A. (2019). Effect of drying on the chemical composition of Çakıldak (cv) hazelnuts during storage. Grasas y Aceites.

[22] (2023). Kenya Bureau of Standards. Hazelnut Kernels—Specification.

[23] Ghirardello D., Contessa C., Valentini N., Zeppa G., Rolle L., Gerbi V., Botta R. (2013). Effect of storage conditions on chemical and physical characteristics of hazelnut (*Corylus avellana* L.). Postharvest Biol. Technol..

[24] Guiné R.P.F., Almeida C.F.F., Correia P.M.R. (2015). Influence of packaging and storage on some properties of hazelnuts. J. Food Meas. Charact..

[25] Razavi R., Maghsoudlou Y., Aalami M., Ghorbani M. (2021). Impact of carboxymethyl cellulose coating enriched with Thymus vulgaris L. extract on physicochemical, microbial, and sensorial properties of fresh hazelnut (*Corylus avellana* L.) during storage. J. Food Process. Preserv..

[26] Correia P., Filipe A., Ferrão A.C., Ramalhosa E., Guiné R.P.F. Effect of moisture on the characteristics of hazelnut kernel during storage. Proceedings of the Livro de Resumos do XVI Encontro de Química dos Alimentos: Bio-Sustentabilidade e Bio-Segurança Alimentar.

[27] Pfeil J.A., Zhao Y., McGorrin R.J. (2024). Chemical composition, phytochemical content, and antioxidant activity of hazelnut (*Corylus avellana* L.) skins from Oregon. LWT.

[28] Pycia K., Kapusta I., Jaworska G. (2020). Changes in Antioxidant Activity, Profile, and Content of Polyphenols and Tocopherols in Common Hazel Seed (*Corylus avellana* L.) Depending on Variety and Harvest Date. Molecules.

[29] Alasalvar C., Karamać M., Amarowicz R., Shahidi F. (2006). Antioxidant and antiradical activities in extracts of hazelnut kernel (*Corylus avellana* L.) and hazelnut green leafy cover. J. Agric. Food Chem..

[30] Król K., Gantner M., Piotrowska A., Hallmann E. (2020). Effect of climate and roasting on polyphenols and tocopherols in the kernels and skin of six hazelnut cultivars (*Corylus avellana* L.). Agriculture.

[31] Ingram L.O., Buttke T.M., Rose A.H., Tempest D.W. (1985). Effects of alcohols on micro-organisms. Advances in Microbial Physiology.

[32] Dobarganes C., Márquez-Ruiz G. (1999). Rancidity in Nuts: Mechanisms and Control. Food Rev. Int..

[33] Gull A., Masoodi F.A., Masoodi L., Gani A., Muzaffar S. (2023). Effect of sodium alginate coatings enriched with α-tocopherol on quality of fresh walnut kernels. Food Chem. Adv..

[34] Hashemi M., Dastjerdi A.M., Shakerardekani A., Mirdehghan S.H. (2021). Effect of alginate coating enriched with Shirazi thyme essential oil on quality of the fresh pistachio (*Pistacia vera* L.). J. Food Sci. Technol..

[35] Habashi R., Zomorodi S., Talaie A., Jari S. (2019). Effects of chitosan coating enriched with thyme essential oil and packaging methods on a postharvest quality of Persian walnut under cold storage. Foods Raw Mater..

[36] Sabaghi M., Maghsoudlou Y., Khomeiri M., Ziaiifar A.M. (2015). Active edible coating from chitosan incorporating green tea extract as an antioxidant and antifungal on fresh walnut kernel. Postharvest Biol. Technol..

[37] Seyhan F., Tijskens L.M.M., Evranuz O. (2002). Modelling temperature and pH dependence of lipase and peroxidase activity in Turkish hazelnuts. J. Food Eng..

[38] Kowalczyk D., Karaś M., Kazimierczak W., Skrzypek T., Wiater A., Bartkowiak A., Basiura-Cembala M. (2025). A Comparative study on the structural, physicochemical, release, and antioxidant properties of sodium casein and gelatin films containing sea buckthorn oil. Polymers.

[39] Kowalczyk D., Karaś M., Kordowska-Wiater M., Skrzypek T., Kazimierczak W. (2023). Inherently acidic films based on chitosan lactate-doped starches and pullulan as carries of nisin: A comparative study of controlled-release and antimicrobial properties. Food Chem..

[40] (2020). Animal and Vegetable Fats and Oils—Determination of Acid Value and Acidity.

[41] (2017). Animal and Vegetable Fats and Oils—Determination of Peroxide Value.

[42] Pegg R.B. (2001). Spectrophotometric Measurement of Secondary Lipid Oxidation Products. Curr. Protoc. Food Anal. Chem..

