# Supplementary material for: Influence of Arabic Gum/Gelatin/Ascorbyl Palmitate Coating on Quality Parameters of Hazelnut Kernels Stored in Plastic Boxes
Source: Molecules. 2025 Oct 19;30(20):4126. doi: 10.3390/molecules30204126 (PMC12565976; doi:10.3390/molecules30204126)
Supplement: Supplementary file 1 [file molecules-30-04126-s001.zip › molecules-3859987-supplementary.pdf]

Table S1. p-values from the F-test for the interaction effect between treatment (control and uncoated hazelnut kernels) and storage time for the analyzed dependent variables.

**Parameter**	**p**
Weight loss	0.037*
Moisture content	0.000*
Hardness	0.889
L*	0.033*
a*	0.333
b*	0.001*
DPPH* scavenging	0.001*
Acid value	0.000*
Peroxide value	0.010*
Thiobarbituric acid reactive substances	0.033*

* p-values < 0.05 (marked with an asterisk) indicate significant interaction effects between treatment and storage time

 https://doi.org/10.3390/molecules30204126
